# EMhub: a web platform for data management and on-the-fly processing in scientific facilities

**DOI:** 10.1107/S2059798324009471

**Published:** 2024-10-07

**Authors:** Jose M. de la Rosa-Trevin, Grigory Sharov, Stefan Fleischmann, Dustin Morado, John C. Bollinger, Darcie J. Miller, Daniel S. Terry, Scott C. Blanchard, Israel S. Fernandez, Marta Carroni

**Affiliations:** a Department of Structural Biology St. Jude Children’s Research Hospital Memphis Tennessee USA; b Medical Research Council Laboratory of Molecular Biology Cambridge United Kingdom; c Department of Biochemistry and Biophysics, Science for Life Laboratory Stockholm University Stockholm Sweden; d Instituto Biofisika–CSIC, Leoia, Spain; National Centre for Biological Sciences-TIFR, India

**Keywords:** data management, web applications, scientific facilities, cryo-electron microscopy, open-source software

## Abstract

Most scientific facilities produce large amounts of heterogeneous data at a rapid pace. Managing users, instruments, reports and invoices presents additional challenges. To address these challenges, EMhub, a web platform designed to support the daily operations and record-keeping of a scientific facility, has been introduced. EMhub enables the easy management of user information, instruments, bookings and projects. The application was initially developed to meet the needs of a cryoEM facility, but its functionality and adaptability have proven to be broad enough to be extended to other data-generating centers. The expansion of EMHub is enabled by the modular nature of its core functionalities. The application allows external processes to be connected via a REST API, automating tasks such as folder creation, user and password generation, and the execution of real-time data-processing pipelines. EMhub has been used for several years at the Swedish National CryoEM Facility and has been installed in the CryoEM center at the Structural Biology Department at St. Jude Children’s Research Hospital. A fully automated single-particle pipeline has been implemented for on-the-fly data processing and analysis. At St. Jude, the X-Ray Crystallography Center and the Single-Molecule Imaging Center have already expanded the platform to support their operational and data-management workflows.

## Abstract 2

This article presents EMhub, a web platform designed to support the daily operations of a scientific facility. EMhub enables the easy management of users, instruments, bookings and project tracking. The application was initially developed to meet the needs of a cryoEM facility, but the versatility of its design makes it suitable for extension to other areas.

## 1. Introduction

In recent decades, the amount and complexity of data across many scientific fields have massively increased. This surge in data has paved the way for new research opportunities and innovative approaches to designing, conducting and evaluating experiments. The biological sciences have also experienced an increase in data generation due to high-throughput instruments and data-collection methods, making biological disciplines more data-driven.

Cryogenic electron microscopy (cryoEM) has experienced a ‘resolution revolution’ thanks to recent technical breakthroughs (Kühlbrandt, 2014*a* ▸,*b* ▸; Bai *et al.*, 2015 ▸). These advancements have led to the determination of larger macromolecular structures at high resolution. As a result, the computational requirements (hardware and software) have reached unprecedented levels. One pivotal breakthrough was the development of direct electron detectors which, together with improved data-acquisition strategies (for example beam image shift), has increased the speed of data acquisition and the volume of generated data (Wu *et al.*, 2015 ▸; Cheng *et al.*, 2018 ▸; Weis & Hagen, 2020 ▸; Cheng & Yu, 2024 ▸). These new detectors can capture movies instead of single images, and they can cover a larger field of view. Furthermore, the high level of automation allows the collection of tens of thousands of movies in a 24 h microscopy session (Bouvette *et al.*, 2022 ▸; Kim *et al.*, 2023 ▸). The combination of larger formats for movie acquisition and faster detector readout has significantly increased the amount of data collected for each cryoEM processing project (Baldwin *et al.*, 2018 ▸).

CryoEM facilities face the challenges of dealing not only with large volumes of data, but also with the complexity of the data and the associated metadata. Different microscopes, cameras and acquisition software can be used, making it difficult to consistently track all ongoing experiments and their parameters. Furthermore, advancements in cryoEM have made the technique more appealing to researchers in other fields, resulting in increased demand and the establishment of many more facilities globally, following two main models (Alewijnse *et al.*, 2017 ▸). The first type is similar to synchrotron facilities used by X-ray crystallographers, providing access to high-end cryoEM instruments for external users. The second type consists of core facilities in research institutions and industry, primarily for the benefit of internal users affiliated with the institution. Some hybrid facilities fall between these two models, serving a mix of users and operational workflows. Data integrity and confidentiality are a high priority for all such facilities, but especially for those serving users from multiple institutions, and particularly for those serving the pharmaceutical industry.

**Figure 1:**
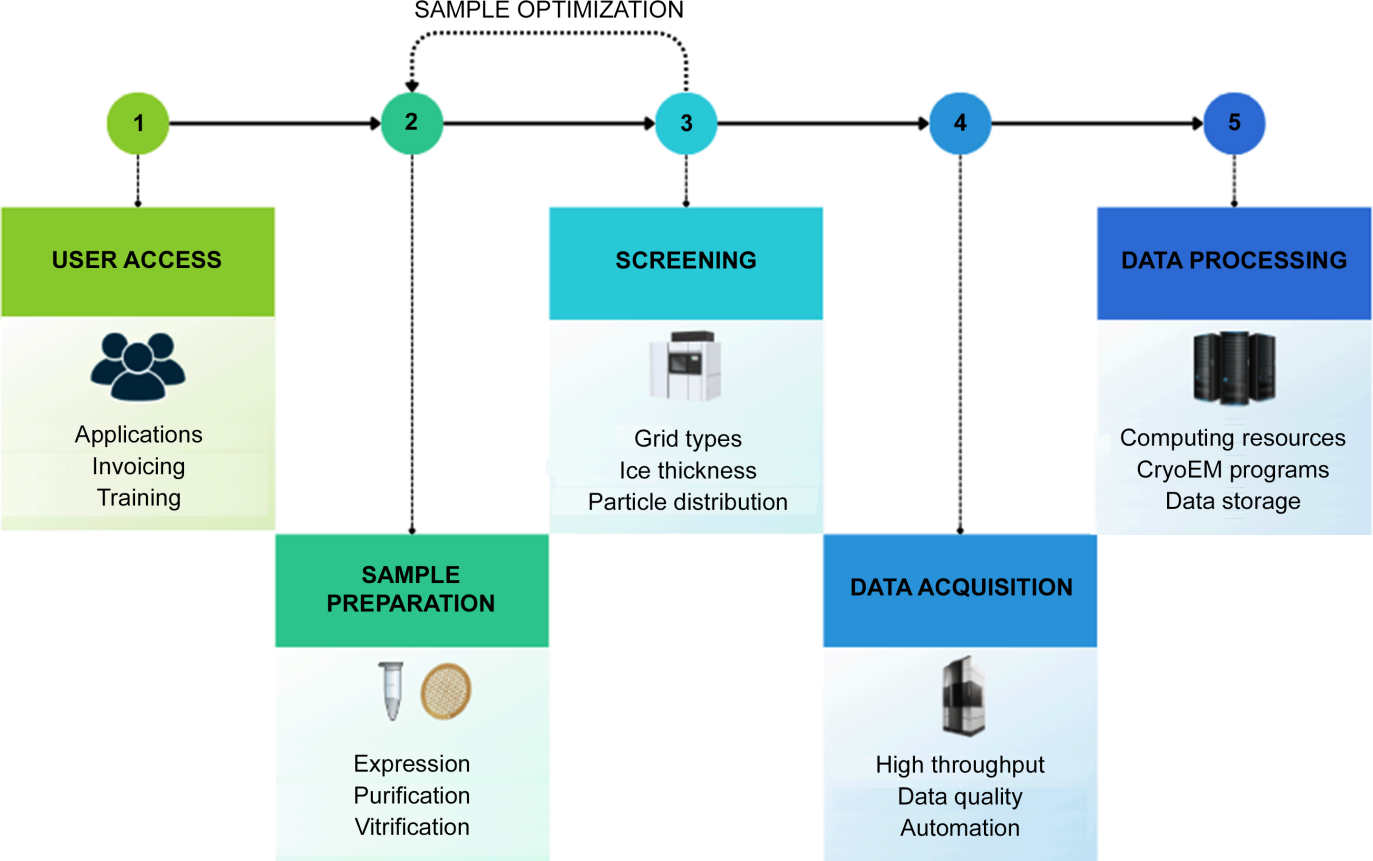
Overview of the data-management workflow for a cryoEM facility. Users request access to the facility and instrument time. Biological samples are prepared, optimized and screened, often requiring iterations and multiple microscopy sessions. Data are acquired using high-end instruments, and some on-the-fly data processing is essential to validate the quality of the data and the performance of the instrument.

Data management is crucial for cryoEM and other scientific facilities. However, many facilities lack proper computational infrastructure and software for their operations. Fig. 1 ▸ illustrates a typical data-management workflow in cryoEM. Access to different instruments must be coordinated and scheduled based on user type, facility policies and the data-acquisition technique. Users may need multiple sessions on several instruments for the same project, and keeping track of related experiments can help with planning and timely execution. Having on-the-fly data-processing workflows is also advantageous for continuously assessing sample and data quality and instrument performance with minimal delay. This helps to ensure efficient instrument usage and high-quality services for users.

In 2017, when the Swedish National CryoEM Facility opened, we used three different web applications and several scripts to manage users, applications and bookings and to conduct on-the-fly processing. As our facility expanded with the purchase of new instruments and an increase in the number of users, the existing solution was unable to meet the growing needs. Generating annual reports and session summaries for invoicing became troublesome and time-consuming. Although we explored applications used by other cryoEM centers, none of them fitted our requirements or were easy enough to customize or expand. As a result, we began to develop EMhub to address our specific needs, and over time it evolved into a more general framework to support various operations. In the following sections we will outline the different components and features of the application.

## 2. Basic concepts and operation

EMhub is a web framework designed to support the daily operations of a scientific facility. It includes functions for user and instrument management, project tracking, invoicing/reporting, and data transfer and processing. The system allows the creation of different types of Users, such as site administrators, facility staff and principal investigators (PIs). Users are typically organized into laboratories by their PI and can also be associated with Applications (for example from different grants, departments or universities).

Staff members or site administrators can define Resources that can be allocated to users and may have an associated cost per Session. These resources can be instruments, such as microscopes, or services. Users will access the resources through the Booking Calendar, following rules set by the facility. Booking rules can be configured per resource and can be defined for different groups of users within the same laboratory or Application. Sessions are carried out by users via an instrument Booking and typically involve data collection for a specific sample. Depending on the user’s experience, a facility manager may be assigned to manage the operation of the Session.

All Sessions and other research experiments can be linked via Projects. One user will be the project owner, while others can be added as collaborators. Within each project, different entries can be created to annotate various events, providing complete traceability and accountability for the research conducted, as well as all relevant parameters. Some types of entries allow the generation of PDF reports for the tasks performed.

### 2.1. Users and Applications

In EMhub, there are four main types of Users: principal investigators (PIs), laboratory members, facility staff (managers) and admin/developers. PIs are independent researchers who run a laboratory and have privileges for laboratory-related operations in the Application. Laboratory members are non-admin users associated with a specific PI, and are the primary unit for invoicing and reporting. They inherit their PI’s booking permissions, such as Applications, booking slots and resource-allocation quota. Facility staff accounts have permission to bypass most rules and restrictions and are intended for use by facility personnel to handle cases that are outside normal operations. Admin/developer roles have higher-level permissions for configuring, maintaining, deploying and troubleshooting the web application, in addition to addressing software operation issues.

Applications are used to organize PIs and their laboratory members within a logical structure. This concept can be adapted to support the allocation of access time and other services in different ways depending on the facility. In national facilities that serve multiple institutes, Applications could be used to represent each external institution. This allows the establishment of distinct booking rules for each instrument and the maintainance of comprehensive statistics. In the case of an internal facility serving users from a single institute or university, Applications can represent different departments or grants associated with PIs. Additionally, Applications have a status property that can be used for various purposes, as the facility chooses. For example, Applications with an ‘active’ status might represent the current ongoing applications in the facility. On the other hand, ‘closed’ Applications might be used for past applications that are no longer active but could be included in reports.

### 2.2. Resources and Bookings

Resources are used in EMhub to represent instruments or services that the facility provides to its users. Examples of instruments in a cryoEM center are microscopes, plunge-freezers and other sample-preparation devices. An example of a service would be regularly scheduled drop-in sessions to offer users support with planning, preparation or data processing for their projects.

Resources are central to the Bookings and time allocation for Applications. Each Resource can have unique booking rules and exceptions for specific Applications. One could establish minimum and/or maximum booking times, cancellation times and session cost per day for a given Resource. For example, a rule might be implemented for a 200 kV screening microscope requiring single-day bookings, while for a high-end 300 kV microscope the rule could entail a minimum Booking of one day and a maximum Booking of two days. Furthermore, session costs could vary for each type of Resource and users may be permitted to book some Resources directly, while approval from facility staff might be required for others.

Specific properties are associated with each Resource, which makes it easier to work with them in the system. These include the ability to define a Resource name, display color, icon and tags. It is also possible to change the status of a Resource from ‘active’ to ‘inactive’ to prevent Bookings or any other operation. Resources can be added to or removed from the Resource List, and the properties of each Resource can also be configured. It is important to note that these actions require the user to have an admin or manager role. More detailed information about Resources can be found at https://3dem.github.io/emdocs/emhub/user_manual/resources_bookings.html#resources.

Bookings are used to manage Resource access based on the rules for each Resource and the laboratory or Application affiliation of a user. One helpful type of Booking is called a ‘slot’, which allows access within a specific date range to certain Applications (user groups), while denying access to others. This feature is particularly useful for a national facility that serves Users from different universities. In this scenario, an Application can be defined for each university. Slots can enable Users from University A to book in one week and Users from University B the following week. This approach allows the facility to allocate overall time efficiently while giving Users the freedom to book Sessions independently according to their own needs.

Only admins or managers can set slots and other particular types of Bookings (for example downtime or maintenance). Recurring events can also be defined, allowing the setup of Bookings for periodically scheduled maintenance or recurring services (for example drop-in sessions every Wednesday). Each Booking has a designated user who is the owner. In most cases, a facility staff member will be assigned to take care of the Session that day. Additionally, an Experiment form can be associated with the Booking, allowing users to provide extra information. This can help the facility staff to plan the required work for that Session more effectively. The Experiment form can be customized to reflect the facility’s workflow and needs.

The Booking Calendar page shows the Bookings for all Resources or a subset of them. The Dashboard page is another common place where users can view Bookings for the current week and past, ongoing or future Sessions. Other pages may also have links to specific Booking dialogs for displaying or editing the information. More information about Bookings canbe found at https://3dem.github.io/emdocs/emhub/user_manual/resources_bookings.html#bookings.

### 2.3. Sessions and Dashboards

Sessions represent the execution of an experiment, typically involving data collection on a specific instrument as per a scheduled Booking. The necessary actions and data management for a Session can vary among facilities. This may involve creating folders or user accounts and transferring and processing data. Sessions have the potential to streamline communication with facility users by providing essential information such as data-collection parameters, data location and instructions for data download on a dedicated page. From the perspective of the facility, Sessions allow the tracking of essential metrics such as the number of Users served and the amount of data collected.

**Figure 2:**
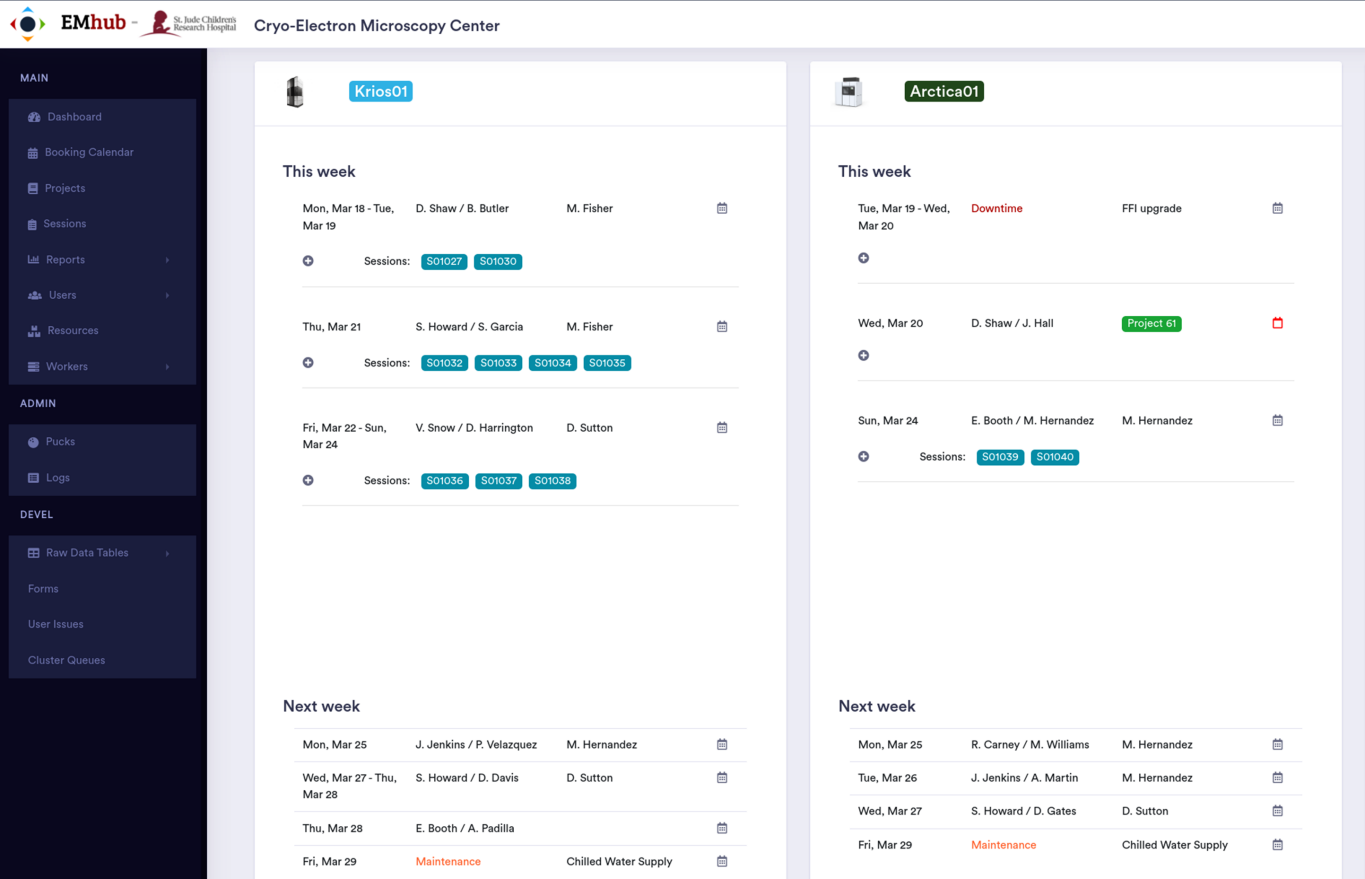
Example of the Dashboard page used at the CryoEM Center in St. Jude Children’s Research Hospital. The page displays Bookings for the current week and allows requests for the next week. These requests are used during the weekly facility meeting to schedule the next Bookings and assign staff.

Even though Bookings can be viewed from the Calendar page, the Dashboard (see Fig. 2 ▸) offers a more focused display. It helps with Session planning by showing the Bookings for each instrument for the current and the following week. This is useful for scheduling instrument usage and planning the staff needed for each session. The EMhub web framework allows the Dashboard page to be customized in order to suit the needs of the facility.

### 2.4. Project and Entries

A Project in EMhub is a valuable way to group and document events (such as Bookings and Sessions) related to a research project over time. Users can see the entire timeline of a Project from its creation. It also enables the collection of various data and metrics related to executed Sessions, such as the number of days of instrument usage, the number of images collected and the total amount of data.

Different types of Entries can be added to a project timeline, providing additional annotations to a project. Using the EMhub configuration, managers can define the available Entries and customize the information for each one. These Entries can be used to perform various operations and later gather information for reporting purposes. More information about Projects and Entries can be found at https://3dem.github.io/emdocs/emhub/user_manual/projects_entries.html.

### 2.5. Reports and Invoices

Reporting and invoicing are crucial aspects for most facilities to manage. While some institutions have a system in place, it may not track instrument usage and data collected in a comprehensive way. EMhub addresses this gap by enabling the generation of detailed Reports and Invoices. Having this information accessible in an organized structure allows further development of tools for analysis and reporting. These tools are essential for demonstrating the performance of a facility to evaluation committees or funding agencies. They can also aid in making informed decisions regarding current and future needs, such as additional personnel or equipment acquisitions.

**Figure 3:**
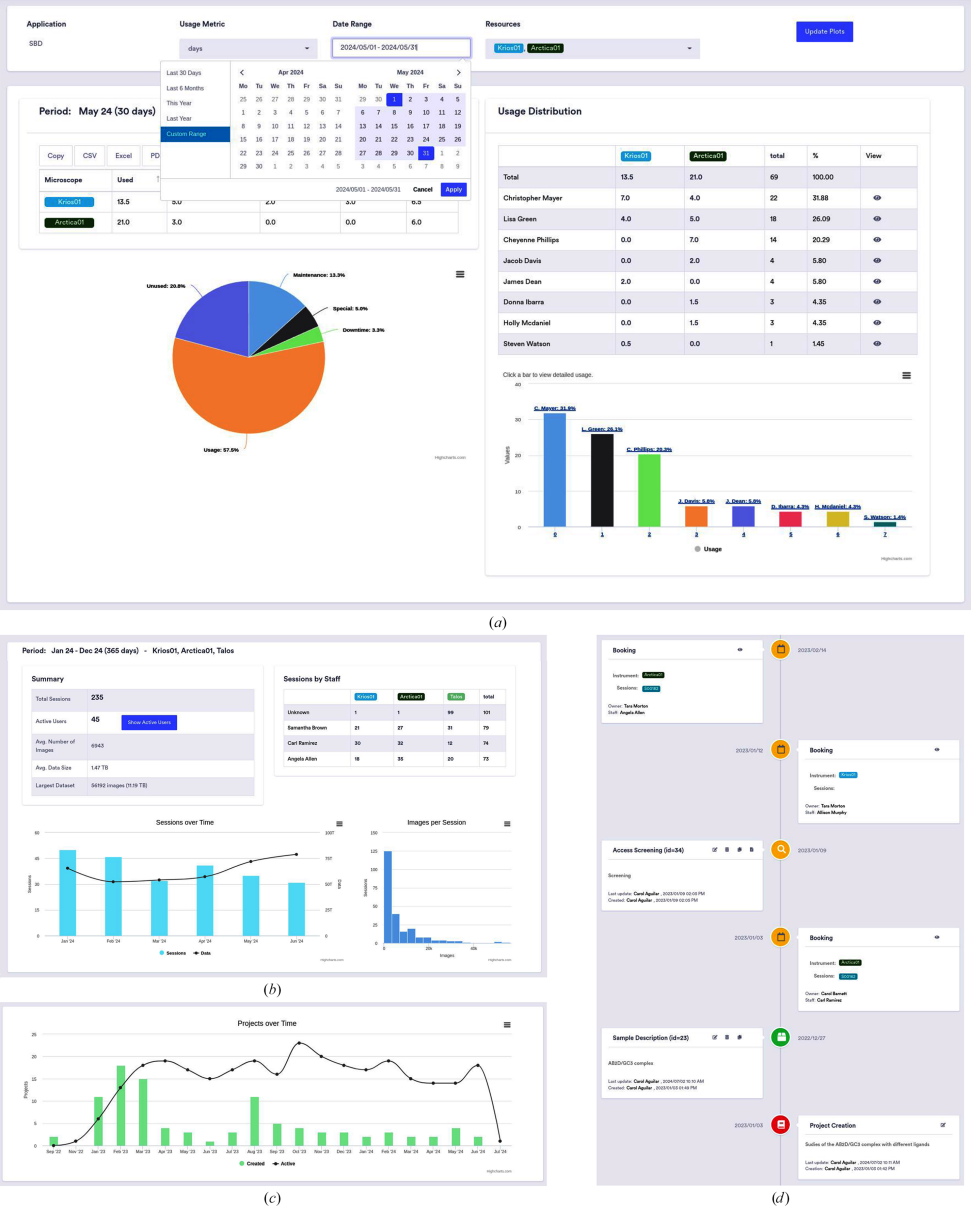
Schematic of the different Reports generated in EMhub. (*a*) Instrument-usage Report for a selected period, grouped by laboratory. (*b*) Overall Report for all Sessions, staff assignments and data collected. (*c*) Total number of Projects and new Projects over time. (*d*) The timeline of a Project showing different types of Entries.

EMhub includes a built-in Report page showing the overall instrument usage, as depicted in Fig. 3 ▸(*a*). Managers can choose which instruments to include in the Report and specify a date range (for example the last month, current year, previous year *etc.*). After updating the plots with the selected parameters, a pie chart displays the instrument usage, downtime and maintenance. Additionally, a list by PIs and/or Applications allows the review of all Bookings contributing to the period under inspection. Fig. 3 ▸(*b*) displays a Session Report over the selected period, showing the number of monthly Sessions, the staff assignment and the amount of data collected. The total number of Projects and newly created Projects can be inspected as shown in Fig. 3 ▸(*c*). The screenshot in Fig. 3 ▸(*d*) shows the different types of Entries in the timeline of a Project.

## 3. Use cases, extensions and software

### 3.1. EMhub at the Swedish National CryoEM Facility

The Swedish National CryoEM Facility provides access to cutting-edge equipment and expertise in single-particle cryoEM, cryo-tomography (cryoET) and MicroED through two nodes belonging to SciLifeLab: one in Stockholm and one in Umeå. At the SciLifeLab node in Stockholm, researchers can perform single-particle cryoEM, MicroED and cryoET for plunge-frozen samples, utilizing a Talos Arctica for sample optimization and two Titan Krios for high-resolution data collection. The node in Stockholm also offers a remote drop-in service for image-processing support. The facility accepts two types of applications: Rapid Access (RA) and Block Allocation Group (BAG). RA applications can be submitted at any time and are evaluated for technical feasibility within the allocated time in the following quarter. BAG applications are evaluated annually by a national Project Evaluation Committee based on scientific merit and technical feasibility.

In the Stockholm node, EMhub has been in use since January 2019. Before that, several software programs were used for data management, as in many other facilities. Initially, there was a web portal where users and PIs registered and submitted applications for instrument access. There was also a separate booking calendar with a different user base unrelated to the web portal. Some custom scripts were also used to create folders for data collection and perform on-the-fly data processing. Furthermore, a significant amount of communication with users occurred via email to provide the necessary details for a session (such as acquisition parameters and data-transfer credentials), and there was an urgent need to document the progress of projects across different sessions.

Several software solutions were considered and evaluated to address these bookkeeping needs. Other facilities at SciLifeLab were using *iLab* (Agilent Technologies; https://www.agilent.com/en/service/laboratory-services/lab-operations-management/core-facilities-management), but this was a commercial product that was unsuitable for modification. *LabArchives* (http://www.labarchive.com/) was used at Stockholm University, but implementing project tracking besides their built-in functionality was cumbersome. *ARIA* (Instruct-ERIC; https://instruct-eric.org/help/about-aria) is management software that is used by some cryoEM facilities, but it is free only for Instruct (https://instruct-eric.org/) countries and some operations seemed to be too complicated for our needs. The Genentech Protein to Structure (Wypych *et al.*, 2021 ▸) information management system is open source, but provides no cryoEM data-processing monitoring tools. None of the evaluated options seemed to be designed with a focus on extensibility and customization.

EMhub was developed to provide a practical solution for all of these needs. It allowed interaction with the portal to import Users and Applications, while centralizing all information under a single system. Access to instruments, Bookings and Sessions are all managed through EMhub. When a Session is created, a corresponding folder and the credentials for data download are automatically generated (based on the User’s application and laboratory). Users can access all of this information on the page of a Session. The facility typically allocates time among different Applications using slots in the calendar. Users from a specific Application can book within the allocated slot days, ensuring fair resource utilization and flexibility for experiment planning. EMhub also provides drop-in services for project support, which can be booked on specified days.

The facility staff use Projects extensively to document the progress of ongoing collaborations. Additionally, numerous Entries related to sample preparation, screening and collection enable tracking of the position of sample grids in the storage dewars (see the online documentation at https://3dem.github.io/emdocs/emhub/user_manual/projects_entries.html#grids-tracking-and-pucks-storage). This allows facility staff to document the screening process for specific grids, so that others can easily access the information during the data-collection session. It also provides research groups with better overall management of the sample-optimization process and enables the smooth transfer of Project responsibility if necessary. Additional levels of permissions and visibility have been implemented in EMhub for Projects of industrial users. General information about these Projects is usually hidden from most users, and it is even possible to limit which facility staff members can access them.

Another feature that is heavily used by the node in Stockholm is the generation of Invoices and Reports. EMhub allows the definition of different Invoice periods (for example quarterly in a year) and accounts for the Sessions in that period for each laboratory. The invoicing information is more transparent for the PIs, who can review all of the Sessions per user in that period and the associated costs. At the end of a period, the facility generates an invoice report that is handed to the financial administrators for actual invoicing. Reports are also crucial for presenting yearly facility numbers and performance for evaluation purposes.

### 3.2. EMhub at the CryoEM Center at St. Jude Children’s Research Hospital

The CryoEM Center in the Department of Structural Biology at St. Jude Children’s Research Hospital provides state-of-the-art instruments and experienced staff scientists to support internal research. The center enables St. Jude researchers to explore intricate biological structures, such as large macromolecular complexes, at almost atomic resolution. A key objective is to offer comprehensive support and training to investigators at all levels across the entire single-particle workflow, from sample preparation to data collection, image processing, 3D reconstruction and modeling. The CryoEM Center is equipped with a 300 kV Titan Krios and a 200 kV Talos Arctica, both fitted with a K3 direct electron detector and a BioQuantum energy filter. The recent installation of another Titan Krios with a Falcon 4 detector and a SelectrisX energy filter has also been completed. The Center also houses advanced sample-preparation, screening and optimization equipment.

Despite not being a national facility, the increase in equipment and the recruitment of new PIs and laboratory members pose some data-management challenges for the center. After some modifications and extensions, EMhub was deployed for daily usage at the center in March 2023. Previously, instrument access was requested via email, with experiment information in a Word document, which was often redundant or inaccurate. All of these processes were moved into EMhub, taking advantage of the flexibility of the Entries in a Project. A new type of Entry (Microscope Access Request) allowed users to request a microscope, specify a desired day and provide information about the sample and help needed for the Session. A new Dashboard page was developed to show all requests for the next week, which could be converted into Bookings and center staff could be assigned. Users can review the assignment after the weekly time allocation and plan accordingly.

Projects have also helped to track the usage of instruments over time in each laboratory, as well as other statistics such as the number of images/data generated per Session and overall in a project. Similarly, reports enable the center to monitor facility operations, especially the usage of microscopes (for example time distribution, downtime, maintenance *etc.*).

### 3.3. Data transfer and on-the-fly processing

Before EMHub, when a data-collection session started at the CryoEM center at St. Jude, some scripts transferred the data to a buffer server and then to the centralized shared filesystem. However, there was no explicit link between the raw images and the booking, which made it challenging to collect overall statistics. With EMhub in place, it became easier to relate the current data collection with the Booking User and their corresponding PI. The transfer script was integrated with EMhub, and the information on transferred files was made available via the Session page.

**Figure 4:**
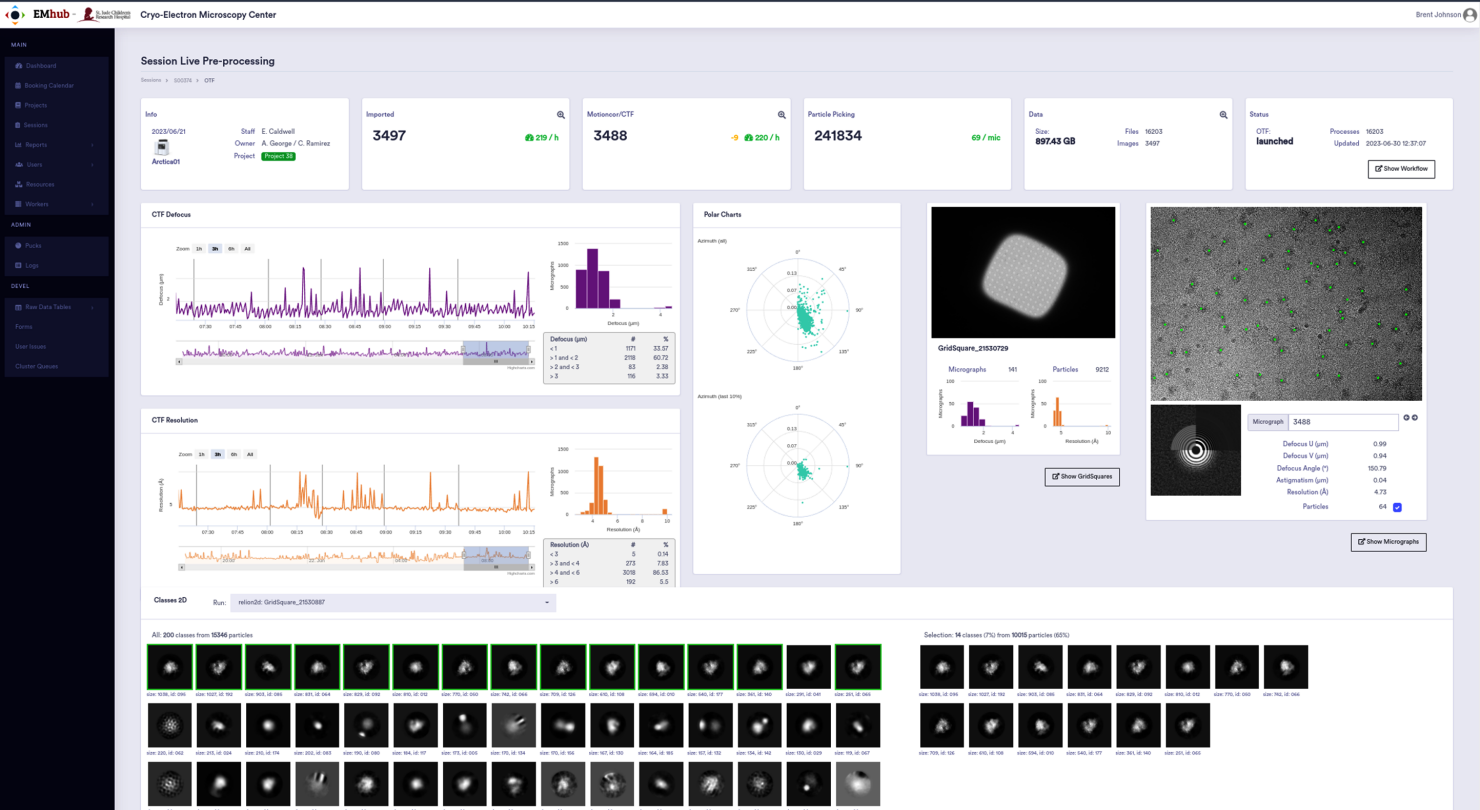
EMhub page to monitor data collection and on-the-fly processing.

For on-the-fly processing, the user manually launched a *RELION* pipeline (Fernandez-Leiro & Scheres, 2017 ▸). The pipeline performed motion correction, contrast transfer function (CTF) estimation (Rohou & Grigorieff, 2015 ▸), blob picking and 2D classification (Scheres, 2012 ▸, 2015 ▸; Kimanius *et al.*, 2016 ▸). Additionally, facility staff generated plots to monitor data quality during acquisition. When EMhub was introduced, one of the initial tasks was to integrate Booking/Session information with the processing pipeline. The existing plots were adapted for the web, and a new Session Live page was created to monitor data-collection progress and processing results, as shown in Fig. 4 ▸. Some input parameters were retrieved from the microscope and data-collection settings, eliminating the need to re-enter them into the pipeline.

To improve our on-the-fly capabilities, we have implemented support for *Scipion* (de la Rosa-Trevín *et al.*, 2016 ▸) workflows as the default pipeline. We have made some modifications to the general processing steps. Firstly, we sped up the motion-correction step using *MotionCor*2 (Zheng *et al.*, 2017 ▸) in batch mode. We then changed the picking process by using the general trained model from *crYOLO* (Wagner *et al.*, 2019 ▸), which has proven to work well for most samples and provides a particle-size estimate. This has allowed us to automate the pipeline without any human intervention. After obtaining the particle size from *crYOLO*, we adjust the box size for extraction and 2D classification accordingly. Another change is the scheduling of 2D classification in batches, where each batch consists of particles extracted from the micrographs of a specific grid square. Upon completing the 2D classification job, we conduct a 2D class selection to gain a rough idea of ‘good classes’ and the underlying ‘good particles’. This approach ensures that a similar number of particles are used as input for each 2D classification job, resulting in quicker completion and providing more real-time feedback about the sample quality. Additionally, we can now gather statistics about the number of good particles per grid square and CTF metrics such as defocus, astigmatism and resolution. This processing pipeline is run automatically for every data collection at St. Jude, and in most cases it provides valuable results. These are kept for internal bookkeeping at the center, but users sometimes request them to continue data processing from that point in their software of choice.

Integrating external workers for tasks such as data transfer or on-the-fly data processing is entirely optional for EMhub. For example, at SciLifeLab only the transfer is used to copy the data to a download server that users can access. At St. Jude, the data are moved to an intermediate staging server and copied later to the group folder in the shared filesystem for the institute. The on-the-fly pipeline is also automatically triggered, and users can monitor it from the internal network. Data-management policies vary from one institution to another, and it is complicated to provide a single solution that accommodates all needs, but EMHub can be configured by each facility with appropriate external workers, including custom-built ones. The workers already implemented in EMhub and the online documentation facilitate the implementation of new workers as required by other facilities.

### 3.4. EMhub for non-cryoEM facilities

#### 3.4.1. Biomolecular X-Ray Crystallography Center

The Biomolecular X-Ray Crystallography Center (XRC) in the St. Jude Department of Structural Biology provides diverse St. Jude researchers with access to tools, facilities and professional assistance for all aspects of high-resolution biomolecular structure determination by X-ray crystallo­graphy. Center staff and trained walk-up users employ robotic instruments for efficient screening of large numbers of crystallization conditions for both soluble and membrane proteins. Along with a host of other instruments, protein crystallization imagers are available for automatic image capture and drop reporting. Once crystals are obtained, they can be assessed via an in-house X-ray diffraction system, and the facility manages external relationships with several synchrotron beamlines, affording St. Jude researchers with regular access to the most advanced light sources and cutting-edge technology.

The XRC has utilized a variety of means to manage access to its on-site resources and the synchrotron time that it stewards. The most recent approach before EMHub involved customized calendars implemented in Microsoft SharePoint, incorporating a variety of custom fields and business rules. Even with these aids, however, the XRC staff spent an undue amount of time coordinating synchrotron time requests and scheduling, much of which took place via e-mail communication, spreadsheets and other documents. Not only was the information that needed to be gathered and collated too diverse to be easily handled via SharePoint, but XRC procedures involve a multi-step, back-and-forth workflow to allocate shared beam time for samples and this was not easily modeled or automated in SharePoint.

In addition to serving well straight out of the box for scheduling access to the on-site instruments at XRC, EMHub proved to be flexible and extensible enough to support the whole beamtime request and approval workflow. Through EMHub, users now provide the needed information about the experiments that they want to perform, XRC staff vet these and allocate available time, users provide essential information about their harvested crystals and the puck in which they are stored, and center staff prepare detailed schedules using a simple drag-and-drop utility and automatically prepare time-sensitive beamline-required documents. Furthermore, all of this information persists in one place, the EMHub database, that can be referenced later to resolve questions ranging from data provenance to distribution of resources. Additionally, XRC staff have found the EMHub REST API, and especially the ability to define custom endpoints, to be useful for automating *post hoc* data-management procedures. This includes the efficient delivery of synchrotron data to institutional storage according to PIs.

#### 3.4.2. Single-Molecule Imaging Center

The mission of the Single-Molecule Imaging Center (SMC) at St. Jude is to make single-molecule imaging methods broadly accessible to scientists at St. Jude and beyond, including those without extensive technical background in microscopy and biophysics. The SMC houses state-of-the-art, custom-built instrumentation for single-molecule fluorescence imaging, including confocal time-correlated single-photon counting (TCSPC) and total internal reflection fluorescence (TIRF) microscopes, as well as ensemble spectrometers, data-analysis workstations and other resources. Much as for cryoEM, single-molecule instruments are expensive to build and maintain. An electronic booking system is essential to ensure judicious use of these valuable resources. Imaging is conducted in custom microfluidic imaging chambers fabricated with careful surface passivisation to avoid nonspecific surface interactions and fluorescent contaminants. These chambers are expensive to fabricate, and thus tracking their availability and performance is essential.

Before EMhub, instrument reservations were managed with a collection of Microsoft Outlook calendars shared with authorized users. This approach was problematic due to the lack of any mechanism to prevent scheduling conflicts, excessively long sessions, making reservations during scheduled downtime or users accidentally modifying other’s bookings. Tracking of microfluidic chambers was handled in a separate Excel document, which suffered from similar issues and also had no direct connection with the booking calendars. EMhub presents a significant improvement on these systems by providing a centralized interface to track instrument bookings and microfluidic chambers that can enforce scheduling rules and associate all of this information together with a project timeline.

In the future, EMhub’s REST API capabilities will be utilized to track standard statistics that measure the health of the instruments and the proper execution of imaging experiments. These include signal-to-noise ratios, particle density, image sharpness (focus), illumination uniformity, optical aberrations, correction factors *etc.* These statistics could be used to determine the timeline of any failures and also as a tool for monitoring users who may need additional guidance or training.

### 3.5. Implementation and software availability

**Figure 5:**
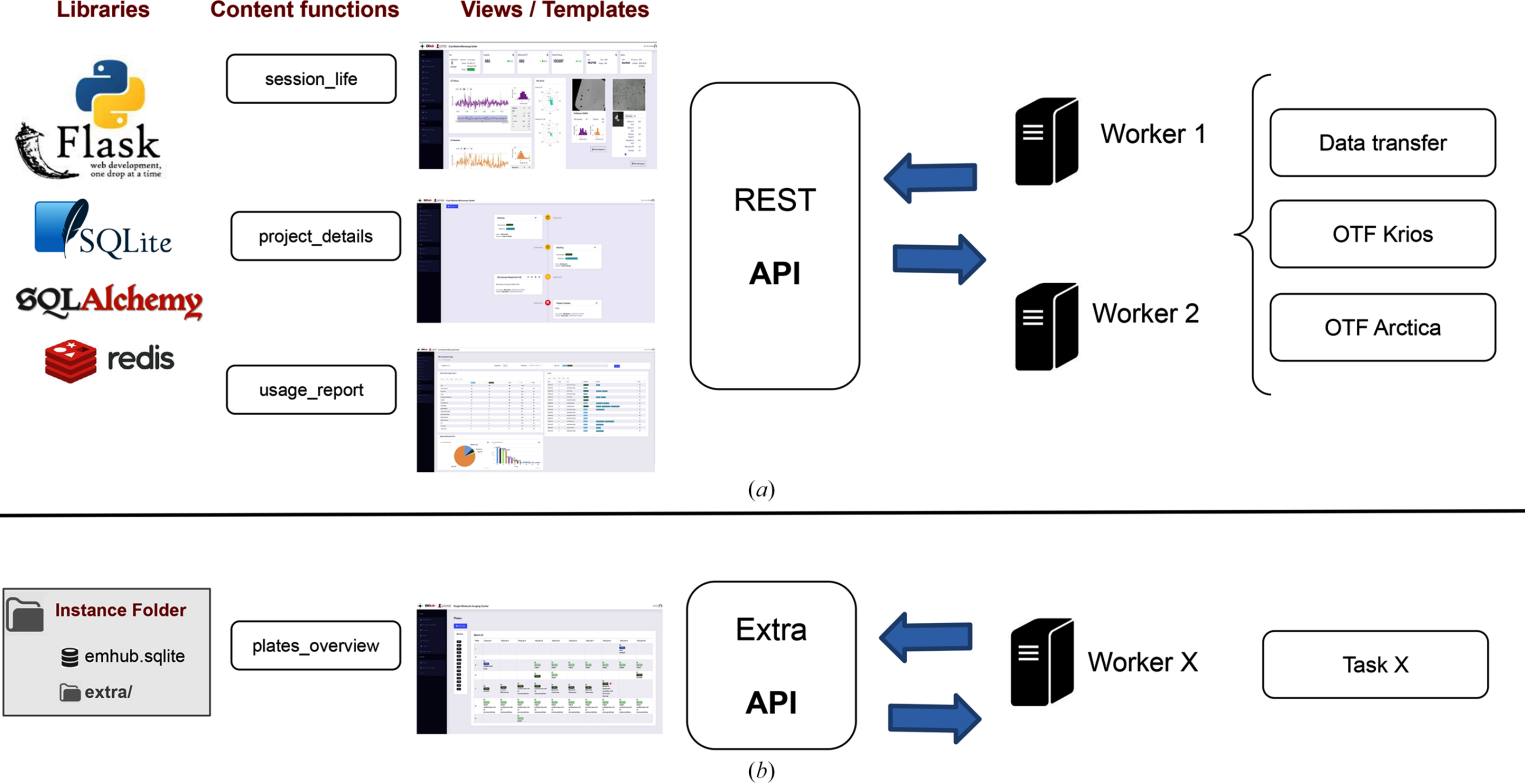
Overview of the EMhub architecture and its customization possibilities. (*a*) The web application is based on Flask and is organized around template/view pages supported by content functions. A REST API allows one to write worker scripts to communicate with the application and execute other tasks. (*b*) Views, content functions and the REST API can be customized and extended by providing an ‘extra’ folder.

EMhub is developed in Python (van Rossum, 2018 ▸) using the Flask (Pallets Project; https://palletsprojects.com/projects/flask/) web micro-framework, which is known for its minimalist design and numerous extension plugins. For data persistence, EMhub utilizes SQLite (Hipp, 2020 ▸) as the database backend through the SqlAlchemy (Bayer, 2012 ▸) mapping layer. A key feature of EMhub is its REST API, which allows other systems to interact with the framework. This REST API has been used to extend the capabilities of EMhub by developing worker scripts that can run independently of the web server to perform tasks such as data transfer or on-the-fly processing. Fig. 5 ▸(*a*) shows an overview of the EMhub architecture. Fig. 5 ▸(*b*) highlights all of the core components that can be extended by providing an ‘extra’ folder inside the EMhub instance directory.

The SQLite database has efficiently handled the current system load, and performance has not declined during its years of operation. Using SqlAlchemy provides flexibility for data operations and will facilitate porting to another database backend in the future if needed. When using workers more real-time communication is required, and for this case we have introduced the Redis (https://redis.io/) in-memory database. EMhub can operate fully without configuring external workers. For example, the on-the-fly cryoEM processing pipeline is an optional module. Currently, *RELION* and *Scipion* pipelines are supported, but it is possible to extend support to others. When using these pipelines, external programs are required for different processing steps. More details about using EMhub with *Scipion* can be found online at https://3dem.github.io/emdocs/emhub/installation/scipion_install.html.

The EMhub web server has modest hardware requirements and can run with few resources. The instance at SciLifeLab runs in a Docker container with a maximum of 8 CPU cores, and memory usage is below 2 GB of RAM memory. At St. Jude, three instances run in a virtual machine with 16 cores, 16 GB of RAM and 16 GB of disk space. The raw data and processing results are not stored in the virtual machine. The on-the-fly processing for cryoEM requires more resources.

EMhub is publicly available under the GPLv3.0 open-source license. Source code can be found at https://github.com/3dem/emhub, together with extensive documentation at https://3dem.github.io/emdocs/emhub.

## 4. Discussion

This article introduces EMhub, a web framework for data management and on-the-fly data processing. The development of EMhub was driven by the challenges faced in the day-to-day operations of a cryoEM facility and the absence of a simple solution to address these needs. The implementation of EMhub has focused on simplicity while supporting user, instrument and booking management tasks. Additionally, it offers comprehensive project and parameter tracking and the generation of reports and invoices. EMhub has already been successfully utilized by the Swedish National CryoEM facility at SciLifeLab in Stockholm and the CryoEM center of the Structural Biology Department at St. Jude Children’s Research Hospital. While having some needs in common, these two facilities have different user types and operational workflows, demonstrating the adaptability of EMhub in diverse scenarios.

The EMhub architecture relies on its REST API, which enables well-defined communication with the system and allows other machines to handle tasks such as account setup, data transfer and on-the-fly processing. The core framework is highly customizable, making it possible to override specific functions or pages and add new features. For instance, the X-ray and Single-Molecule Centers have expanded the core EMhub framework to suit their needs. Several features are already planned in the immediate and medium-term future of the development roadmap. For example, we could extend sample management in EMhub to include more information such as shipping, biosafety and storage. As another example, a simple news mechanism can be implemented that will help facility staff inform users about instruments, training or any other relevant information. A ticket system could also help to handle different tasks and allow users to report problems or suggestions. Implementing a worker that runs on the cluster could enable the development of a page to monitor the status of nodes in the cluster and submitted jobs.

Additionally, we plan to implement more tools to analyze the results of cryoEM on-the-fly processing; for example, filtering micrographs or particles based on different criteria such as CTF parameters. This will allow users to reuse the provided results to continue their data-processing pipeline more efficiently. Another future direction is to extend EMhub to integrate with cryoET data processing. We can reuse some of the infrastructure developed for single particles and expand existing functionalities to visualize and analyze cryoET data. The timeline for implementing these and other features will depend on the need and the human power to address them.

The current implementation of EMhub has already proven to be valuable, and we expect it to appeal to other facilities as well. Since different facilities have diverse needs and operations, we do not think that a single monolithic solution can be ideal for every case. Accordingly, we have focused on developing a framework with many useful built-in features that can be extended and customized for different situations. Nonetheless, some programming skills are required, such as the basics of the Python language and web programming concepts (HTML, Javascript, REST *etc.*). Moreover, the data-management workflow can also be complex in some cases, which needs to be considered when integrating into EMhub. To facilitate this process, we have put a lot of effort into documenting EMhub (https://3dem.github.io/emdocs/emhub/developer_guide/), providing many examples that could serve as a guide when implementing new features. We envision EMhub becoming a community hub where other developers will implement custom features while contributing back with core functionality or enhancements. We plan to support this collaborative development model to grow EMhub to serve the scientific community better.
